# Cutaneous Implications of Liver Cirrhosis: A Case of Acrodermatitis Enteropathica

**DOI:** 10.7759/cureus.82734

**Published:** 2025-04-21

**Authors:** Sameera Shuaibi, Najat AlSejari, Samer Farhud, Jeanne Nguyen, Caley McIntyre

**Affiliations:** 1 Internal Medicine, Ochsner Clinic Foundation, New Orleans, USA; 2 Internal Medicine, Jaber Hospital, Kuwait City, KWT

**Keywords:** acrodermatitis, acrodermatitis enteropathica, liver cirrhosis, liver failure, zinc deficiency

## Abstract

Acrodermatitis enteropathica (AE) is characterized by a constellation of symptoms that result from a deficiency in serum zinc levels. The commonest cause of AE is hereditary autosomal recessive genetic mutations that impair zinc absorption in the small intestine, while acquired causes come second. Liver disease has been implicated in the development of acquired zinc deficiency. Herein, we present a case of AE with severe exfoliative skin lesions that occurred secondary to decompensated liver cirrhosis.

## Introduction

Zinc is an important essential trace element that plays multiple roles in wound healing, DNA synthesis, enzymatic reactions, and immune functioning, along with other vital functions [1]. Zinc deficiency can either be hereditary or acquired [2]. It is often overlooked when the diagnosis of skin rashes in acute hospital settings is required. Physicians tend to evaluate more common causes of skin rashes before reaching the conclusion of acrodermatitis enteropathica (AE). From a dermatological perspective, it initially manifests with tender scaly rashes that can progress to more severe presentations such as bulla formation in cases of extremely low serum levels of zinc [3]. An important organ that plays a crucial role in zinc metabolism is the liver. Liver failure can interfere with zinc metabolism due to a multitude of reasons, namely, hypoalbuminemia, induction of cytokines, and increased bodily excretion. Conversely, low zinc levels can worsen hepatitis via its diminished anti-oxidant properties [4]. This report sheds light on the unfortunate dermatological sequelae, AE, that occurred in the setting of zinc deficiency secondary to deteriorating liver failure.

This article was previously presented as a meeting poster/abstract at the 2024 American College of Gastroenterology Annual Scientific Meeting and Postgraduate Course on October 28, 2024.

## Case presentation

**Figure 1:**
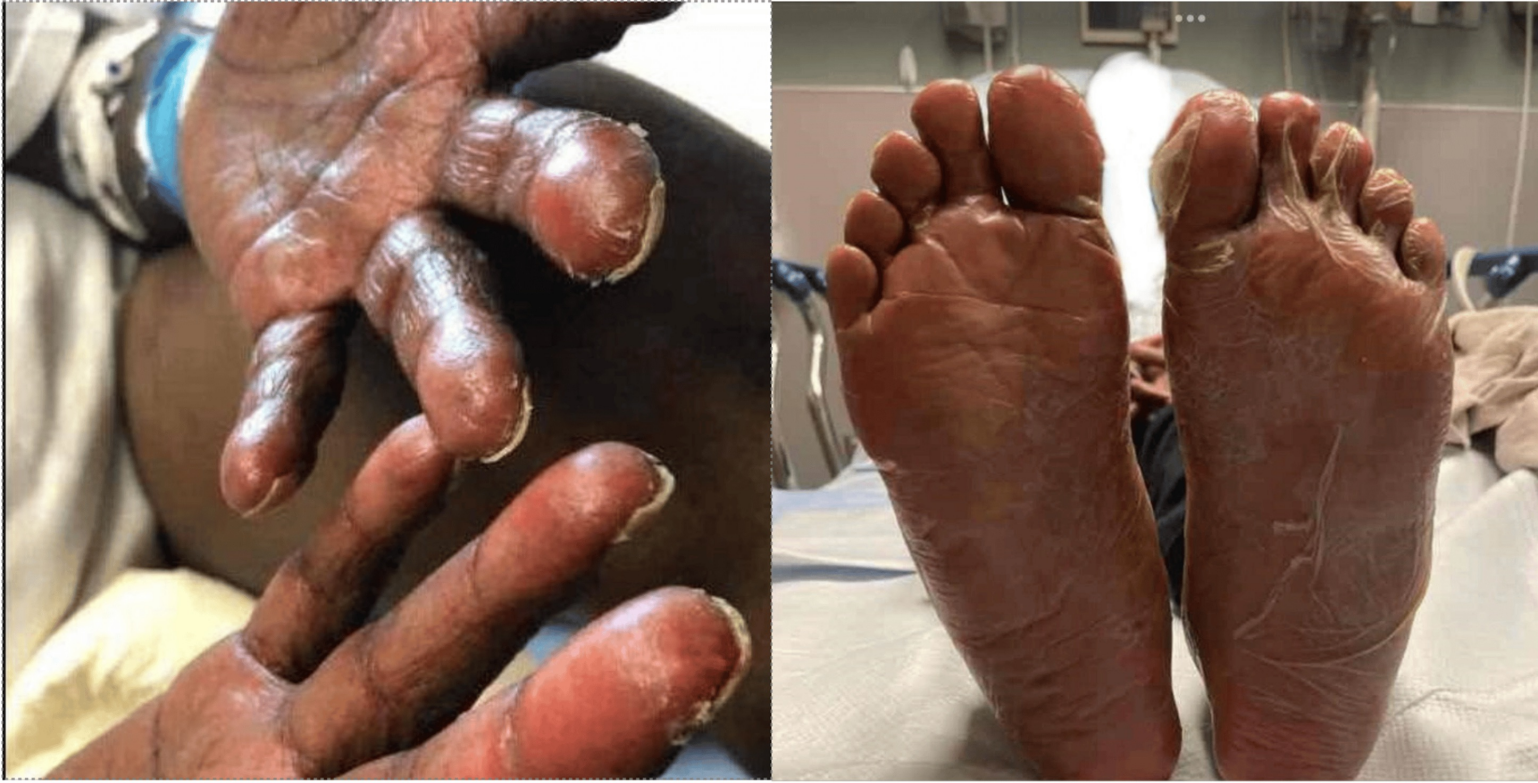
Acrodermatitis enteropathica desquamative rash confined to the patient's palms and soles. A desquamative rash, marked by skin peeling, was limited to her palms and soles during the initial episode of liver deterioration.

**Figure 2:**
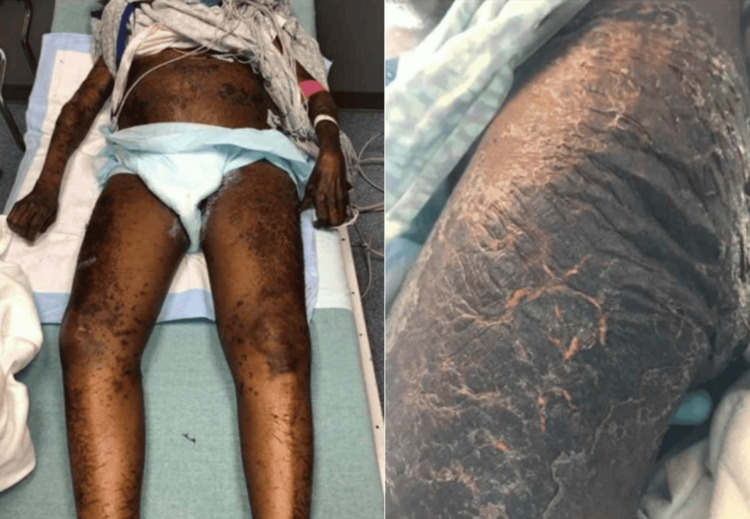
Acrodermatitis enteropathica lesions spread throughout patient's body. Widespread acrodermatitis enteropathica lesions occupying the entire body of the patient during her subsequent deterioration in liver function (that was followed by her ICU admission).

**Figure 3:**
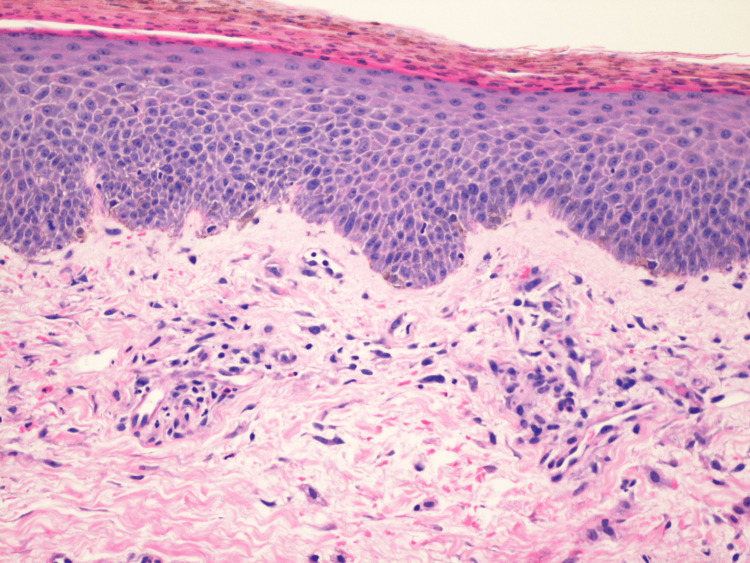
Histopathological evidence of AE obtained from a biopsy specimen of the thighs. Sections show confluent parakeratosis with pigment deposition, with the granular layer being focally absent. There is irregular epidermal acanthosis, focal pallor in some areas of the upper epidermis, and a cluster of vacuolated cells within the upper epidermis. Within the underlying superficial dermis, there is a mild perivascular lymphohistiocytic infiltrate with rarer neutrophils. Erythrocyte extravasation is also noted within the superficial dermis.

A 49-year-old female with a history of alcoholic liver disease and stage 5 chronic kidney disease presented to the ED from her rehab center with worsening widespread desquamative rash (Figure 1). It was confined to peeling at the level of her palms and soles. Upon admission, she was assessed by the dermatology team who started her on IV ceftriaxone and IV vancomycin, which improved her rash, and the patient was discharged home later. Soon after, she was readmitted directly to the ICU for worsening hepatic encephalopathy and rash. Her AST and ALT levels at that time were 99 U/L and 60 U/L, respectively. She reported that the rash had since spread to her extremities, abdomen, and back, and started to form on her neck/chest but denied any mucosal involvement (Figure 2). The dermatology team was re-consulted, and suspicion of AE was among the top differentials this time around. The use of antibiotics during the previous admission appeared to temporarily improve the presumed secondary skin infection, but the underlying dermatologic condition persisted and progressed. Thus, as part of the broad workup, zinc levels were ordered and found to be 46 mcg/dL (normal range: 70-120 mcg/dL), indicating a significant deficiency. The progression of liver dysfunction, evidenced by a rise in total bilirubin from 10.7 to 24.2 mg/dL and AST elevation to 275 U/L, paralleled the worsening dermatologic findings, suggesting that hepatic impairment exacerbated zinc depletion. This prompted the start of high-dose oral zinc supplementation due to suspicion of AE (Table 1). Consequently, the dermatology team opted for a biopsy of her rash from her thighs (Figures 2, 3). In the meantime, due to her ongoing liver disease and malabsorption, it was seen best fit to treat her zinc deficiency while she was inpatient via the parenteral route instead of orally with aggressive local wound care to prevent complications from widespread desquamation. Pathology specimen of the thighs was consistent with AE. Meanwhile, her hospitalization was complicated by severe hypoxia that she could not recover from and she deceased. For this reason, it was not possible to assess the effects of aggressive zinc supplementation on her skin condition.

**Table 1:** Lab values prior to ICU admission and during ICU stay. Laboratory values correlating to her presentation just prior to her ICU admission and subsequently during her ICU hospitalization. Liver function was notably worsening. During that time, the dermatology team requested zinc levels. WBC, white blood cell; HGB, hemoglobin; PLT, platelet; INR, international normalized ratio; AST, aspartate aminotransferase; ALT, alanine transaminase; ALP, alkaline phosphatase

Laboratory test	Initial labs (prior to ICU)	Subsequent labs (ICU)
WBC	21.13 K/uL	22.34 K/uL
HGB	7.5 g/dL	7.1 g/dL
PLT	77 K/uL	172 K/uL
INR	2.1	2.0
AST	99 U/L	275 U/L
ALT	60 U/L	85 U/L
ALP	140 U/L	166 U/L
Total bilirubin	10.7 mg/dL	24.2 mg/dL
Total protein	5.2 g/dL	5.6 g/dL
Zinc	-	46 μg/dL

## Discussion

Multiple pathophysiological mechanisms have been observed behind zinc deficiency in the background of advanced liver disease. The liver plays an important role in zinc homeostasis; hence, zinc deficiency may occur as a result of advanced liver disease. A very basic example would be via decreased nutritional intake (mainly proteins) in cirrhotic patients which can interfere with zinc metabolism. It is worthy to mention that albumin is the main carrier of zinc in the body; thus, inadequate albumin can lead to zinc deficiency by affecting its transport [5]. Other factors include diminished hepatic extraction, effects of cytokines (IL-6), and the inherent effect of alcohol itself in those with alcoholic liver disease, which can interfere with zinc absorption [6]. The catabolic state of liver cirrhosis in itself can induce a substantial loss of zinc in urine [7]. Additionally, patients with liver cirrhosis are usually placed on a diuretic regimen to offload the fluid buildup/edema in the body, and as such, these means lead to increased loss of both zinc and albumin in the urine, further accentuating the deficiency of zinc [8-9].

Not only does the liver interfere with zinc absorption, but low zinc levels also can interfere with proper liver functioning. In fact, many of the manifestations of liver disease, such as poor appetite, loss of body hair, testicular atrophy, immune dysfunction, and hepatic encephalopathy, are caused in part due to impaired zinc metabolism. This is to point out that zinc is an important cofactor in multiple enzymatic reactions including sex hormone synthesis and urea cycle/nitrogen metabolism [10], leading to testicular atrophy and hepatic encephalopathy, respectively. As liver disease progresses and zinc stores are depleted, ammonia levels inversely rise [7]. To confirm this pathophysiological process, some studies have also shown benefit in the mental status of patients when zinc supplementation is added to hepatic encephalopathy patients [11]. Additionally, zinc acts as an anti-oxidant combating oxidative stress. In ongoing hepatic injury and inflammation, zinc deficiency may alter its anti-oxidative properties, thereby leading to unopposed inflammation (hepatitis) [12].

The skin is the third largest organ containing zinc; hence, cutaneous manifestations are expected with zinc deficiency, ranging from mild to severe, with erythematous scaly plaques and eczematous or vesiculobullous lesions, depending on how severe the zinc deficiency is. Nail, oral, and ocular changes, such as onychodystrophy, stomatitis, and conjunctivitis, can also be seen [13]. In regards to distribution, skin manifestations typically occur in the perioral, acral, and perineal regions. Deficiency of zinc can lead to impaired wound healing, disrupted immune responses, inhibition of cell growth and division, and subsequent inflammation manifesting initially as eczema and progressing to vesiculobullous lesions. This was seen in our patient who developed worsening and continued spread of her skin manifestations with declining liver function at that time. Of note, due to impaired immunity as a result of zinc deficiency, infections may occur. This could have been the reason behind the fact that the patient’s skin manifestations initially improved after her initial presentation as superimposed skin infection may have existed at that time.

**Figure 4:**
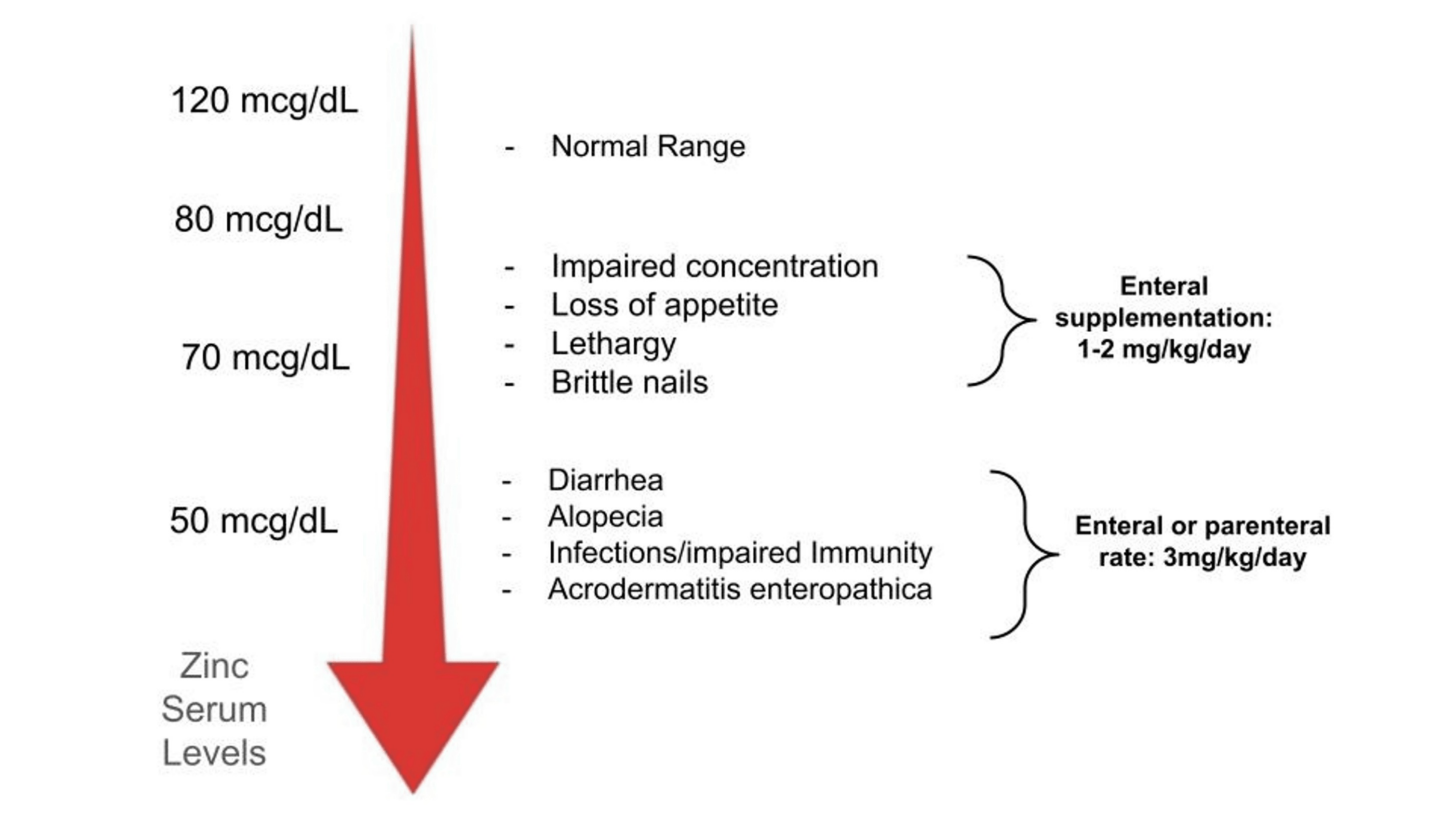
Serum zinc concentrations and correlating symptoms and modes of repletion. Zinc deficiency is associated with a plethora of manifestations. The severity and character of symptoms depend on the level of zinc. In case with severely reduced zinc levels, parenteral correction with higher doses of zinc may be required for repletion.

Diagnosing AE can be extremely challenging due to the overlap of AE with other skin diseases and the fact that AE lesions can be diverse, occurring altogether and not necessarily following the perioral/acral/perineal distribution. As a result, many patients with AE may witness progression of their lesions due to delays in diagnosis and treatment. When clinical suspicion arises, zinc serum levels can help support the diagnosis that could be confirmed with a skin biopsy, as in our case. From thereon, depending on the severity of the deficiency, zinc replacement is initiated. Particularly in AE when skin lesions are advanced, zinc supplementation should be started at a rate of 3mg/kg/day either enterally or parenterally depending on the overall condition of the patient (Figure 4). 

## Conclusions

In conclusion, AE is a rare skin condition that occurs in the setting of zinc deficiency. It is often associated with conditions such as advanced liver disease. Regarding our patient, zinc deficiency was a likely major contributor to her dermatologic findings, although concurrent factors such as infection and liver-related skin manifestations (i.e. hepatic dermatosis) cannot be fully excluded. As such, early diagnosis and treatment are deemed vital to lessen the impact of zinc deficiency.

This case highlights the importance of considering AE in patients with liver cirrhosis and unexplained dermatologic findings, especially when nutritional deficiencies are likely. Although the patient’s clinical course precluded assessment of therapeutic response, earlier recognition and zinc supplementation might have altered the outcome.
